# On the use of the h-index in evaluating chemical research

**DOI:** 10.1186/1752-153X-7-132

**Published:** 2013-07-30

**Authors:** Rosaria Ciriminna, Mario Pagliaro

**Affiliations:** 1 Istituto per lo Studio dei Materiali Nanostrutturati, CNR, via U. La Malfa 153, 90146 Palermo, Italy

**Keywords:** Bibliometrics, Impact factor, H index, Chemical research, H index chemists

## Abstract

**Background:**

The h index bibliometric indicator for evaluating scientists and scientific institutions plays an increasingly important role in evaluating contemporary scientific research, including chemistry.

**Results:**

Citations are meaningful. The best way of measuring performance is to use the informed peer review, where peers judge on the base of a bibliometric report, once the limits and advantages of bibliometric indicators have been thoroughly understood.

**Conclusions:**

Expanded and improved use of bibliometric indicators such as the h index in a useful and wise manner is suggested.

## Background

In a 1980 article [1] analyzing the performance of a large number of chemists at American universities bibliometric experts concluded that “Publications measure productivity, citations the usefulness of the publications, and citations/paper the relative extent to which groups of papers generate interest in the scientific community”. Thirty years later, tenure and promotion committees do not use the simple citations/paper ratio anymore. To evaluate candidates they rather increasingly use the “*h*-index”, namely the number h of publications with at least h citations introduced by Hirsch in 2005 [2]. For example, a chemist with an h-index of 25, has published 25 papers that have each received at least 25 citations.

Publications with the greatest impact are those having at least h citations (the “Hirsch core”). A typical value for a successful scientist is an h value of 20 for 20 years of research; an outstanding scientist will have h = 40 for 20 years in science. Given its reliance on most cited papers, the index is clearly biased towards age. Hirsch therefore called for its normalisation for age by dividing the index by the number of years since the appearance of the first publication, affording the “m quotient”.

The h index, a natural number, is approximately proportional to the square root of the total citation counts, and linearly proportional to the total number of publications, [3] combining the number of papers (quantity) and the number of citations (quality). In the words of Bornmann, [4] an academic cannot have a high h-index without publishing a substantial number of highly cited papers, as the index favours those who publish a continuous stream of papers with lasting impact.

To get a higher h index, an individual needs at least 2 h+1 extra citations. For example, to increase the index from 4 to 5, at least 9 citations are needed. The higher the h index the more citations are required to increase it. It means that the difference between higher h index values (25 and 26, for example) is much greater than between lower values (6 and 7, for example).

Measuring quality of scientific research is if course important, especially today when many countries adopt research policies that emphasize excellence and have implemented evaluation systems to identify top researchers [5]. A great variability still exists on the importance accorded by department heads and committees to the h index and related metrics, and letters of recommendation by peers, are often a useful means placing these metrics within a broader context of research impact and efficacy. Richard Zare, former department chair at Stanford University’s Chemistry Department, for example, wrote that the department collects 10–15 recommendation letters from outside experts prior to the tenure decision [6]. Yet, when open positions are made available through the Internet from institutions competing for the best scientists from all over the world, interested scientists apply submitting their *curricula*, often highligthing the h index on the very first page of the CV.

Does the overall number of citations received exceed 2,972? Then our candidate will rightly claim to be among the most cited 1% of chemists in the world, since among the 22 scientific disciplines listed in the citation thresholds of Thomson Reuters*’ Essential Science Indicators,*[7] this was the threshold to be among the most cited 1% chemists during the decade ending on April 2011.

Indeed, despite diffuse criticism (see below), the use of bibliometric indicators to assess the quality of applicants has become widespread at promotion committees and funding agencies. Research chemists, and scientific evaluators, need therefore to understand more closely the origin, the limitations and the virtues of these indicators in contemporary chemical research.

### The impact factor and the h index

Following the 1955 concomitant foundation of the Institute for Scientific Information in Philadelphia and the publication of a seminal paper in *Science*, [8] in 1961 Eugene Garfield, a chemist turned into a linguist and bibliometric expert, started to calculate the journal impact factor (IF) as a tool to assess the quality of a scientific publication, namely as a a metric for the comparison of journals within a specific subject category [9]. Instead than counting the number of articles a journal published in previous year, the new “Science Citation Index” started to rank journals through the IF index, namely the average number of times articles from the journal published in the past two years have been cited in the *Journal Citation Reports* year.

For example the IF of a *Advanced Synthesis* &*Catalysis* in 2012 is calculated by dividing the number of citations in the *Journal Citation Reports* in 2012 by the total number of articles published by the journal in 2011 and in 2010. The tool, despite criticism for which a high journal impact factor can be the result of many citations of a few papers rather than the average level of the majority, [10] has become the main yardstick to assess the quality of scientific journals.

In 1992, the Institute of Scientific Information was acquired by Thomson Scientific & Healthcare, whose latter parent company (Thomson Corporation) in 2008 bought also Reuters (an information company based in the UK). Today, the Canadian multinational information firm Thomson Reuters continues to publish the *Journal Citation Reports*, an annual publication including the IF values of most scientific journals eagerly waited for each June by publishers as well as by researchers interested in publishing their research in high-IF journals, and get promoted.

This situation has attracted fierce criticism, including a recent international petition [11] calling on the world scientific community to eliminate the role of the journal impact factor in evaluating research for funding, hiring and promotion. However, one may notice, that the very same scientists more often criticizing this system are those who have extensively published their work in high-IF journals.

After all they, too, are interested to give their research broad visibility, as scholarship requires effective communication, [12] and the effectiveness of communication lies in the feedback it generates. For example, Petsko, a widely published genetist, expressed his fierce criticism writing in the high IF (9.04) open access journal *Genome Biology*[13]:

«… The impact factor of where you publish becomes a surrogate for the use of your own judgment. No one bothers to read anyone's papers when they're up for a fellowship or being considered for a job or for a promotion or having their grant proposal evaluated; all you do is look to see how many papers they've published in high-impact journals.

«No one considers whether the work was better suited to a more specialized journal or a journal where other work that puts it in context was published previously; no one considers whether those handful of high impact-factor journals have the best referees or whether they in fact may have a disproportionate number of incorrect papers because of the pressure to publish there.

«And look, over reliance on one stupid number gave a small bunch of editors enormous power over the careers of people who, for the most part, they never met or heard speak, and whose body of work they never read.»

Indeed, life scientists were not only critical, but also rather creative. Open access journals such as *PLoS Med* and *PLoS Biology* in 2012 had high and growing IF values of 16.27 and 11.45. Yet, the publisher emphasizes [14] that articles in all journals should be assessed on their own merits rather than on the basis of the journal in which they were published. The same publisher thus initiated a program to provide a growing set of measures and indicators of impact at the article level that includes citation metrics, usage statistics, blogosphere coverage, social bookmarks and expert assessment.

In this uneasy context, physicist Jorge Hirsch in 2005 introduced [2] the h index to measure the cumulative impact of a researcher’s output exactly by looking at the amount of citations her/his work has received. Now, in place of the total number of papers or of the total number of citations, a single natural number defined as the number of a scientist’s N_p_ papers having at least h citations each corrects for papers that are *not* cited.

The limitations of the index, too, are well known. The h index can be applied to researchers in the same field, and should be used to compare researchers of the same age. It does *not* take into account the number of authors on any given paper and it is biased towards researchers writing numerous review articles.

Another drawback is that, being a natural number, it has low resolution, and a relatively narrow range so that it is common for a group of scientists to have an identical h-index. Zhang solved both these problems by introducing in 2009 the e-index, a real number that complements the h-index for the ignored excess citations [15]. Most software applications enabling fast calculation of the h index, today include the e-index, too.

The h-index of a single publication, too, can be easily calculated, and correlated with peer assessments of manuscripts. For example, the analysis of a total of 1,814 manuscripts reviewed by referees of *Angewandte Chemie International Edition* in the year 2000, [16] clearly showed that after publication manuscripts with positive ratings by the referees show higher h index values than manuscripts with negative ratings (and later published elsewhere). It may therefore come as no surprise to learn that Thomson Reuters today includes the h-index of journals as part of its new “Citation Report Index”, making it an accepted measure of academic achievement.

The index eventually became the tool for “evaluating an individual”, [2] despite a *caveat* from Hirsch himself that “it can never give more than a rough approximation to an individual’s multifaceted profile, and many other factors should be considered in combination… especially in life-changing decision such as the granting or denying of tenure” [2].

### Calculating the h index

The calculation of a scientist’s h index requires citation data. In general, today the search of the number of publications and citations listed for individual scientists in the available literature databases is simple [17]. Four comprehensive databases, all belonging to private organizations, are normally employed by selection committees evaluating chemists. The fee-based databases Web of Science (from Thomson Reuters, which offers also the Book Citation Index and the Conference Proceeding Citation Indexes), Scopus (from Elsevier), and Chemical Abstracts (American Chemical Society); [18] and the freely available Google Scholar.

It should be noted that no chemistry-specific server of peer-reviewed articles exists (such as PubMed Central in biomedicine or arXiv in physics). Chemists remain, by far, the most conservative scientists towards open access (OA) publishing, namely towards the option to publish their research in peer-reviewed journals that make their content freely and permanently available on the World Wide Web. Only 181 out of 9,417 OA journals are devoted to chemistry, namely less than 2% [19]. In other words, chemists remain bound to a reward system strongly based on citations and (high) journal IF values, lagging at least five years behind life scientists [20]. Once accepted by leading researchers in chemistry, the OA model of publication would instead maximize the impact of chemical research, as it happened for research in life sciences.

Fee-based conventional systems only include citation to journal articles (and not to books, book chapters and conference papers) and include citations in journals that are listed in their own databases. For example, the “Web of Science” covers more than 12,000 journals, with coverage from the 1970s. Scopus instead claims to cover “nearly 18,000 titles from more than 5,000 publishers”. Needless perhaps to say, both encourage publications in journals indexed in their own databases.

Google Scholar, on the other hand, comprehensively records all citations including books, conference papers, teaching materials and working papers, often returning material which is scholar. It is interesting to read a biochemist insight, comparing the virtues and limitations of the aforementioned databases [21]:

«Looking at my most cited paper, which has been cited 367 times (Google Scholar) or 267 times (Web of Science) or 287 times (Scopus) I found that Google Scholar included 11 Chinese articles, 10 book chapters, 15 theses, 4 patents, 1 blog, 1 grant application, and 6 mysteries. Eliminating these 48 still leaves 319.

«Quite a bit higher than Web of Science and Scopus, probably because Google counts citations from articles that are still in press (my Neurobiology of Aging paper was published online but “in press” for 23 months, during which citations could be tracked in Scholar but not Web of Science). This is probably also why Google Scholar counts 17 citations (16 “normal”) of my most recent paper whereas Web of Science only counts 9 – many of these citing articles were recently published.

«So should Chinese articles be excluded? Are book chapters irrelevant? Theses, well, no one reads theses so maybe there’s a bit of inflation there. I do think it’s a sign of impact when a blog, grant, or patent refers to your paper and believe that these things should be included in the citation counts».

This inclusiveness and especially the free nature of Google Scholar make of it the database of choice for most researchers worldwide, even if it has many and important disadvantages, especially in the field of chemistry. For example, Bornmann and co-workers in 2009 examined 1837 papers published in chemistry, mostly in the journal *Angewandte Chemie*, and found that although Google Scholar retrieved 95.1% of the articles, its total citation counts were only a fraction (21%) of Web of Science citation counts, mainly because Google Scholar returned zero citations for half of the sample [22]. However, Google programmers follow scientific literature and constantly upgrade their algorithm. Hence in early 2013 Harzing reported [23] results showing that coverage of Google Scholar in chemistry had improved considerably over the years, being now at a level where chemistry can be included in comparative searches, especially for summary metrics such as the h-index.

Scientists go to the Google Scholar web page (http://scholar.google.com) or download and install the *Publish or Perish*[24] software. Following voluntary registration, Google Scholar allows researchers to manually add their research output and then associate all their previous papers with the identifier.

In both cases, covering an undisclosed and frequently updated base of online data, [25] the Google’s secret algorithm rapidly provides the outcome of the search, including citation statistics (h index, overall number of citations) and, in the case of Harzing’s software also the e index score, and times cited per year since publication.

The researcher then starts to polish the data by erasing papers by scientists with the same name or, conversely, manually add papers published under different names; as well as to cancel questionable academic material from the citations list of each publication. After this editing activity is complete, a reliable updated value h index is obtained.

### Use the h index to evaluate researchers?

Citations in chemistry are meaningful. Already in 1980, data for a large number of chemists at American universities clearly led to this conclusion [1]. The h index alone, however, cannot render the multidimensional complexity of research performance. For example, the multidisciplinary nature of a candidate’s research should be acknowledged and rewarded, as the boundaries that have separated the traditional chemistry disciplines in the 20^th^ century -- inorganic, organic, organometallic, solid state, (bio)polymer and materials chemistry -- have broken down to create one large multidisciplinary community with a keen scientific and technological interest in all aspects of the chemistry. Has perhaps the candidate published her/his research in a large number of journals devoted to once separate domains of chemical research? Such a feature should be inserted in open faculty position announcements, and rewarded accordingly.

Science, however, is about progressing knowledge [26]. And the essence of scholarship is communication. Hence, practical aspects such as the ability to attract funds, the number of managed projects and tasks, activity in public outreach and so on, should not enter serious scientific evaluation. Especially in countries, like Italy, that are known for academic cronyism, [27] bibliometric indicators should be the main parameters used to assess performance in scientific research. In other words, the best way of measuring performance is to use the informed peer review, where peers judge on the base of a bibliometric report. It may be relevant here to notice that in Italy the new system for appointing University professors since mid 2012 includes an habilitation which is based only on bibliometric indicators, [28] and not on the discretional analysis of the CV made by panel members who can easily act complacently.

## Conclusions

Instead than eliminating altogether the use of bibliometric indicators, [10] such as the h index or the impact factor, we agree with a critical approach to expand and improve their use in a useful and wise manner.

For example, Bornmann and Marx recently advanced [29] recommendations for a set of standard indicators for evaluating researchers. In alternative to the h index, they propose to use the number of publications for a researcher which belong to the 10% of the most-cited publications in their field and publication year (P_top 10%_) [30]. Based on the percentile approach, this indicator takes into account successful publications normalised for time *and* field. An example taken from their original work vividly renders the concept.

Table 1 shows the publications of three researchers, two with a similar long career (>30 years since the first publication), and one with considerably shorter academic age. The age-normalised m quotient already reveals a clear advantage in the performance of Researcher 2 (m=2.5) compared to Researcher 1 (m=1.7) and Researcher 3 (m=1.2).

**Table 1:** Overview of the scientific performance of three researchers

**Indicator**	**Person 1**	**Person 2**	**Person 3**
**Productivity**			
Article	143	54	43
Editorial	1	1	4
Letter	3	0	1
Meeting Abstract	3	0	2
News Item	0	2	0
Note	12	0	1
Proceedings Paper	26	17	40
Review	2	2	4
Total publications	190	76	95
Number of articles, notes, proceedings papers and reviews	183	73	88
Number of publications as first author^*^	15	17	38
Number of publications with no co-authors^*^	0	5	12
Year of first publication^*^	1980	2001	1981
Number of years between the first publication and 2011^*^	32	11	31
Number of publications per year (arithmetic average^*^	5.9	6.9	3.2
**Impact**			
Total citations^**^	15,192	3,796	7,828
Number of citations per publication^**^ (arithmetic average)	83	52	89
Proportion of self-citations in total citations^*^	3.4%	6%	5.8%
Average percentile (median)^**^	15.9	6.2	8.3
h index^**^	54	27	38
m quotient^**^	1.7	2.5	1.2
P_top 10%_^**^	70	31	48
PP_top 10%_^**^	39.3%	52.5%	57.8%
P_top 10%_ quotient^**^	2.2	2.8	1.6

Reproduced from Ref. [28], with kind permission.

Remarks:

^*^ Based on publications of all document types.

^**^ Based on articles, letters, reviews, notes and proceedings papers.

Even though the h index is age-normalised to give the m quotient, the second step, normalisation for field is missing. Bornmann and Marx therefore use the age-normalised P_top 10%_ indicator.

The P_top 10%_ quotient for Researcher 1 is 2.2. The normalised value of 2.8 for Researcher 2 shows that she/he has published around twice as many P_top 10%_ as Researcher 3 (P_top 10%_ quotient=1.6).

In conclusion, practitioners of chemical research should not refrain from mastering the use of bibliometrics indicators. In the Internet era, rapid access to reliable bibliometric data has become possible at virtually no cost. Hence, basic education on scientometrics should be included in the *curricula* of undergraduate chemistry students in order to let future researchers to proactively use statistical data describing their research, as well as to access the achievements of others. This, *inter alia*, will allow them to better present their own achievements, as well as to better understand the state and the evolution of a certain research field.

Research chemists can, and should, periodically upload online bibliographic data describing their research (for example on Google Scholar, but also on other online platforms such as ResearchGate), which will provide them with periodic feedback about those who are citing and using their research. Evaluators, in their turn, have in advanced and yet simple indicators such as the aforementioned P_top 10%_ quotient an informative and synthetic parameter offering far better information than the simple h index. Why, in conclusion, should we research chemists be scared by numbers and information?

## Methods

Data and software applications were accessed via the Internet. Extensive researches were carried out in the scientific literature dealing with scientometrics, research evaluation and scientific publishing, particularly in the field of chemistry.

## Abbreviations

IF: Impact factor; OA: Open access.

## Competing interests

The authors declare that they have no competing interests.

## Authors’ contributions

RC carried out the preliminary research on h index in chemistry, participated in the thoughtful discussions and helped to draft the manuscript. MP conceived of the study, and drafted the manuscript. Both authors read and approved the final manuscript.
